# Ergogenic Aids to Improve Physical Performance in Female Athletes: A Systematic Review with Meta-Analysis

**DOI:** 10.3390/nu15010081

**Published:** 2022-12-24

**Authors:** Olga López-Torres, Celia Rodríguez-Longobardo, Raquel Capel-Escoriza, Valentín E. Fernández-Elías

**Affiliations:** 1 Faculty of Sport Sciences, Universidad Europea de Madrid, 28670 Madrid, Spain; 2 Social Sciences of Physical Activity, Sport and Leisure Department, Faculty of Physical Activity and Sport Sciences, Universidad Politécnica de Madrid, 28040 Madrid, Spain; 3 Raquel Wellness Instituto de Nutrición y Salud, 28050 Madrid, Spain

**Keywords:** women, sports, performance, supplementation, exercise, nutrition

## Abstract

Most intervention studies investigating the effects of ergogenic aids (EAs) on sports performance have been carried out in the male population. Thus, the aim of this systematic review and meta-analysis was to summarize the effects in the existing literature of EAs used by female athletes on performance. A literature research was conducted, and a descriptive analysis of the articles included in the systematic review was carried out. Meta-analyses could be performed on 32 of the included articles, evaluating performance in strength, sprint, and cardiovascular capacity. A random-effects model and the standardized mean differences (SMD) ± 95% confidence intervals (CI) were reported. The results showed that caffeine helped to improve jumping performance, isometric strength values, and the number of repetitions until failure. Caffeine and sodium phosphate helped to improve sprint performance. Aerobic tests could be improved with the use of taurine, caffeine, and beta-alanine. No conclusive effects of beetroot juice, polyphenols, or creatine in improving aerobic performance were shown. In terms of anaerobic variables, both caffeine and sodium phosphate could help to improve repeated sprint ability. More studies are needed in female athletes that measure the effects of different EAs on sports performance, such as beetroot juice, beta-alanine or sodium phosphate, as the studies to date are scarce and there are many types of EA that need to be further considered in this population, such as creatine and taurine.

## 1. Introduction

The ingestion of ergogenic aids (EA) to enhance performance has become popular in recent years and is a widely used strategy by athletes [1]. Although the terms “EA” and “nutritional supplementation” are used interchangeably in the literature, we can differentiate certain nuances between them. Nutritional supplementation would encompass sources of nutrients or other substances with a physiological or nutritional effect that complement the normal diet [2]. EA, on the other hand, include any short- and/or long-term nutritional practice that seeks to improve sports performance, to enhance training adaptations and/or to help post-exercise recovery [3]. Therefore, all EA are nutritional supplements, but not all supplements are EA.

There are a plethora of sports EAs on the market that aim to achieve different indirect performance improvements, such as a modulation of the inflammatory response [4], reducing oxidative stress [5], improving homeostasis recovery [6], adaptation of signaling pathways [7], reducing fatigue [8], or improving aerobic [9] and short, high-intensity exercise performance [10]. For some of these EAs there is solid proven scientific evidence (e.g., caffeine (CAF), creatine (CRE), nitrate, beta-alanine (BA), and bicarbonate (SB)) [11,12] while for others there is a lack of evidence or it is very limited and inconclusive (e.g., the amino acid N-acetylcysteine or polyphenols (PPs)) [12].

One of the most used EAs is CAF due to its effect on improving force production, decreased ratings of perceived exertion and pain, and improvements in aerobic capacity, among others [13,14]. CRE is another widely used EA to improve performance in short, high-intensity exercises [15], with consistent scientific evidence. L-carnitine, on the contrary, although broadly used, lacks conclusive scientific studies that prove its ergogenic effect [16]. The conclusive literature on the use of new emerging EAs such as PPs is not very extensive [17,18], although their effect as a nutritional supplement to improve health in the non-active population has been already demonstrated [19,20,21].

Some studies of recreational and elite athletes have shown some differences in the consumption levels of nutritional supplements and EAs in swimmers [22] and rugby players [23], while in other sports, such as handball [24], rowing [25], or fencing [26] no differences between consumption levels were found. Regarding gender differences in EA consumption, studies have mostly found a similar prevalence in the use of EA among males and females [27]. Despite this similar prevalence in EA consumption between male and female athletes, most intervention studies investigating the effects of EAs in sports performance have been carried out in the male population, with few studies showing the effects of different types of EAs in female athletes and sportswomen [13,28,29].

Due to the widely proven physiological and hormonal differences between men and women that cause the processes of absorption, distribution, metabolism, and disposal of pharmacological substances to differ [30,31], the response to EAs may not be the same. Some studies show dissimilar responses by gender to different EAs, although these are scarce [32,33,34]. For instance, females could present lower changes in heart rate but higher blood pressure changes than males when consuming CAF [32]. Furthermore, they need a smaller amount of BA than men to reach the same relative increase in muscle carnosine levels [34]. Another difference is that women present higher NO_2_ levels and have a more oxidative type of skeletal muscle than men, so they may benefit more from the consumption of NO_3_ containing EAs such as beetroot juice (BJ) [28]. These differences could influence sports performance, but more research is needed to better understand the specific effects of EAs in females.

It is therefore necessary to compile information about intervention studies using EAs in physically active females and sportswomen, in order to synthesize the positive, neutral, or negative effects they produce. Hence, the objective of this systematic review and meta-analysis was to summarize the effects in the existing literature of EAs used by female athletes and recreationally practitioners on performance.

## 2. Materials and Methods

A systematic review of the literature and meta-analysis were carried out following the PRISMA guidelines and the PICOS criterion. This work was registered in PROSPERO (CRD42022340185).

### 2.1. Search Strategy and Study Selection

A literature search was conducted during April–June 2022 using the PubMed and Web of Science databases. A search strategy was devised using the words “female” OR “women” AND “sport” OR “exercise” AND “ergogenic aids” by including them in the “topic” section in Web of Science and in the Title/Abstract section in PubMed. The research was filtered by date, including articles from the last 15 years. No language filter was applied.

The inclusion criteria were intervention studies on female athletes or recreationally active women using EAs to enhance their physical performance, both acutely and longitudinally. By physical performance we mean the skills deemed necessary for increased success and a reduced risk of occurrence/severity of injury when exercising and/or practicing sports (i.e., muscle strength, sprinting capacity, cardiorespiratory fitness, and all their magnitudes and components). Studies with women over 18 years old were included with no age limit. The exclusion criteria were studies that solely included measurements on parameters other than sports performance, studies which included the effects of EAs in both men and women without comparing the results by sex, and articles in which the participants used supplements without seeking ergogenic support. Conference proceedings, doctoral theses, dissertations, case studies, and other reviews and meta-analyses were excluded.

**Figure 1:**
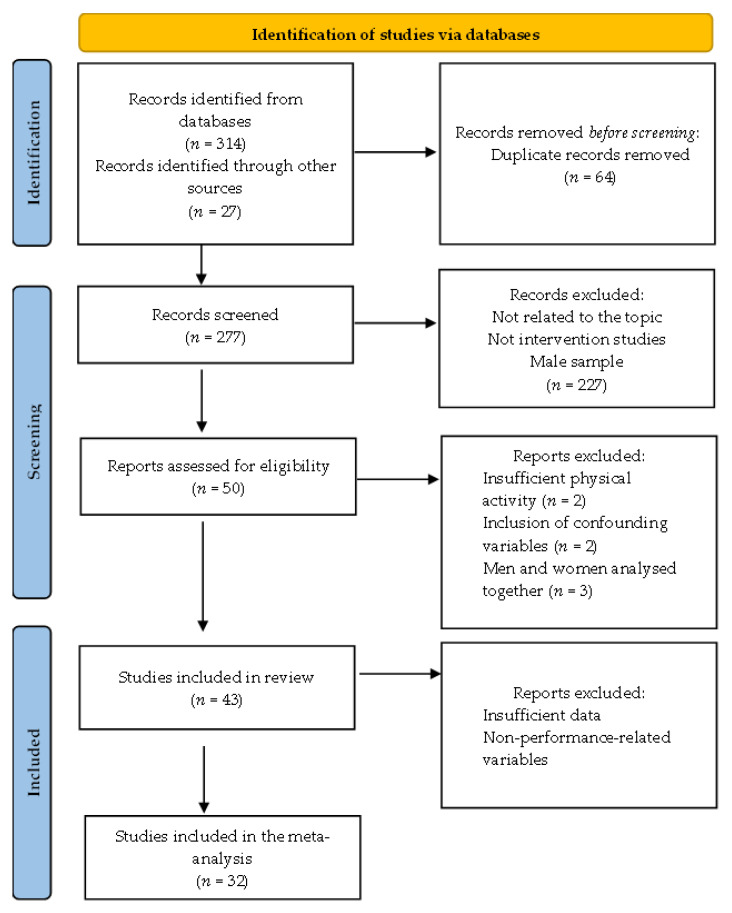
Flow chart for study selection according to PRISMA criterion.

Initially, 341 items were identified. After eliminating duplicates and filtering according to the inclusion criteria, 43 articles were included in the review and 32 in the meta-analysis. The study selection flow chart is shown in Figure 1.

### 2.2. Data Extraction

The selection of articles and the data extraction on study source, study design, study quality, participants’ sample size, participants’ characteristics, ergogenic substances, and performance outcomes of the interventions were conducted by three independent reviewers (CRL, OLT, and RCE). If disagreements appeared, a third author (VEF-E) was included, and the discrepancies were resolved through discussion. Trials were not excluded based on quality. A priori, the main outcomes analyzed were changes in the measures of physical performance triggered by the ingestion of EA. All the evaluations were performed in duplicate, independent of each other. Disagreements in the analysis were resolved through consensus. 

### 2.3. Risk of Bias

The risk of bias assessment was independently evaluated by two reviewers with the PEDro scale [35]. This tool provides information about the allocation and randomization process, the blinding process, the data obtained, and the statistical analysis. The PEDro score scale considers a rate of 0–3 ‘poor’, 4–5 ‘fair’, 6–8 ‘good’, and 9–10 ‘excellent’. Furthermore, a total PEDro score of 8/10 is considered optimal for trials assessing complex interventions (e.g., exercise).

### 2.4. Statistical Analysis

To analyze the data, the studies were separated according to the physical abilities examined. All the variables that assessed the same physical capacity were analyzed together. Thus, an analysis was carried out to determine performance in strength (divided into jumping and power, isometric strength, and endurance strength), sprint, and cardiovascular capacity (aerobic and anaerobic).

Of the 43 articles selected in the review, 11 were discarded for the meta-analysis because they did not include sufficient data, or they analyzed other types of variables not related to exercise performance. Therefore, measurements were taken from the EA intake groups and the placebo groups to make a comparison among them. The most representative variable of the physical capacity was included in the data analysis. In those studies that included two EAs, the two corresponding variables analyzed independently with respect to the placebo group were added.

The outcome measure to carry out the analysis was the standardized mean difference (SMD). A random-effects model was then fitted to the data. The restricted maximum-likelihood estimator (RML) [36] was used to calculate the amount of heterogeneity (i.e., tau^2^). In addition to the RML estimator, the Q-test for heterogeneity [37] and the I^2^ statistic are detailed. If any amount of heterogeneity was noticed (i.e., tau^2^ > 0, regardless of the Q-test’s results), a prediction interval for the true outcomes was also given. To investigate whether studies might be outliers and/or influential in the context of the model, studentized residuals and Cook’s distances were utilized. Studies with values greater than the 100 × (1 − 0.05/(2 × k))th percentile of a standard normal distribution were considered to be potential outliers (i.e., utilizing a Bonferroni correction with two-sided alpha = 0.05 for k studies included in the meta-analysis). Studies which presented a Cook’s distance bigger than the median plus six times the interquartile range of the Cook’s distances were considered influential. The rank correlation test and the regression test were applied to test for funnel plot asymmetry, making use of the standard error of the observed results as a predictor. To perform all the analyses, the free statistical software Jamovi was used (version 1.6.15) [38].

## 3. Results

The characteristics of the studies are presented in Table 1.

The articles included dated from 2008 to 2022. Most studies were cross-sectional in nature (*n* = 35, 81.4%). The remaining studies incorporated interventions evaluating the longitudinal effects of EAs. Thirty-five out of the forty-three studies had a cross-over design (81.4%) and the remaining eight articles included a specific control group.

A total sample of *n* = 710 women was included in the qualitative analysis with a mean age of 26.5 years and a mean BMI of 22.7 kg/m^2^.

Of the 43 studies, 20 (46.5%) were carried out with team sports players (*n* = 339), 10 with endurance trained athletes (23.3%, *n* = 144), 6 with resistance trained athletes (14.0% *n* = 82), 4 with recreationally active females (9.3%, *n* = 75), 1 with sports students (2.3%, *n* = 24), 1 with tennis players (2.3%, *n* = 17) and 1 with kayak athletes (2.3%, *n* = 5) (see Table 1).

Regarding the EAs used to enhance performance, CAF was used alone in 18 studies, (41.9%) [10,29,39,40,41,42,43,44,45,46,47,48,49,50,51,52,53,54], combined with BJ in 2 articles [55,56], and with taurine (TAU) [57] and sodium phosphate (SP) [58] in another 2 studies. BA was presented alone in five studies [59,60,61,62,63] and combined with CRE in one study [64]. CRE was utilized in three studies [65,66,67]. The use of BJ alone appeared in four articles [28,68,69,70] and citrulline malate (CM) in another two [71,72]. Another combination was SP + BJ [73]. Isolated articles were also identified using TAU [74], SB [75], sodium citrate (SC) [76], PP [77], and purslane (PUR) [78].

### 3.1. Quantitative Analysis

The results are presented divided into the three physical capacities analyzed: strength, sprint, and cardiorespiratory performance. The variables were added independently of the EA used in order to see which EA produced the greatest increase in performance and on what specific test.

#### 3.1.1. Strength

- Power and jumping performance

**Figure 2:**
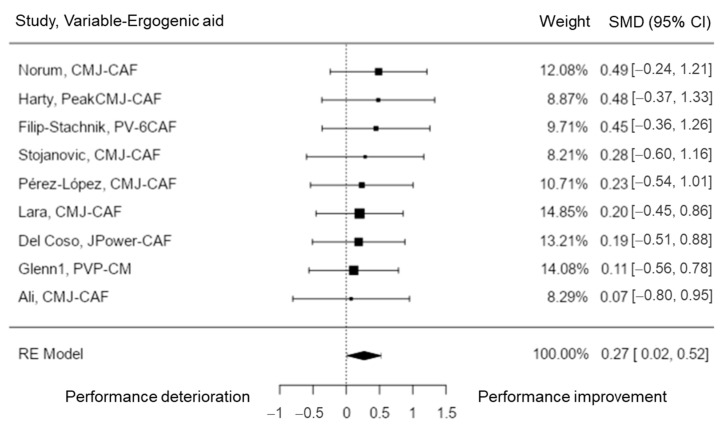
Power and jumping performance forest plot. CMJ = counter movement jump; PV = peak velocity; JPower = jumping power; PVP = peak vertical power.

This capacity was analyzed in nine studies [29,39,40,43,46,51,52,53,71]. The SMD varied from 0.07 to 0.49, with most of the estimates being positive (100%). According to the random-effects model, the estimated average SMD was \hat{\mu} = 0.27 (95% CI: 0.02 to 0.52). Hence, the average outcome was significantly different from zero (z = 2.09, *p* = 0.0.037). There was no significant amount of heterogeneity in the true outcomes according to the Q-test (Q (8) = 1.28, *p* = 0.996, tau^2^ = 0.00, I^2^ = 0.00%). An exploration of the studentized residuals revealed that none of the studies had a value larger than ± 2.77 and therefore there was no indication of outliers in the context of this model. Considering the Cook’s distances, none of the studies could be overly influential (Figure 2). Neither the rank correlation nor the regression test indicated any funnel plot asymmetry (*p* = 0.477 and *p* = 0.735, respectively). Thus, the results showed that CAF helped to improve countermovement jump height and other jumping performance variables.

- Isometric strength

**Figure 3:**
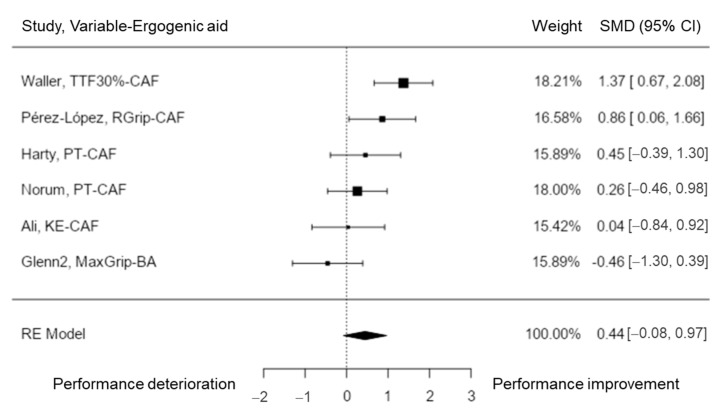
Isometric strength performance forest plot. TTF = time-to-task failure (30% maximal voluntary contraction); RGrip = right hand grip; PT = peak torque; KE = knee extensor.

Isometric strength was analyzed in six studies [40,43,47,51,52,60]. The observed SMD ranged from −0.46 to 1.37, with most estimates being positive (83%). According to the random-effects model, the estimated average SMD was \hat{\mu} = 0.44 (95% CI: −0.08 to 0.97). Hence, the average outcome did not differ significantly from zero (z = 1.67, *p* = 0.094). Considering the Q-test, the true outcomes appear to be heterogeneous (Q(5) = 13.02, *p* = 0.023, tau^2^ = 0.26, I^2^ = 61.13%). A 95% prediction interval for the true outcomes ranged between −0.68 and 1.57. Thus, even though the average outcome is taken to be positive, in some studies the true outcome may be negative. None of the variables had a value larger than ± 2.64 according to an examination of the studentized residuals, and therefore there was no presence of outliers in this model. Based on the Cook’s distances, none of the variables could be overly influential (Figure 3). Neither the rank correlation nor the regression test indicated any funnel plot asymmetry (*p* = 0.136 and *p* = 0.119, respectively). Therefore, the results showed that CAF could help to enhance time-to-task failure, grip strength, and peak torque values. No effects of BA on grip strength could be shown.

- Resistance strength

**Figure 4:**
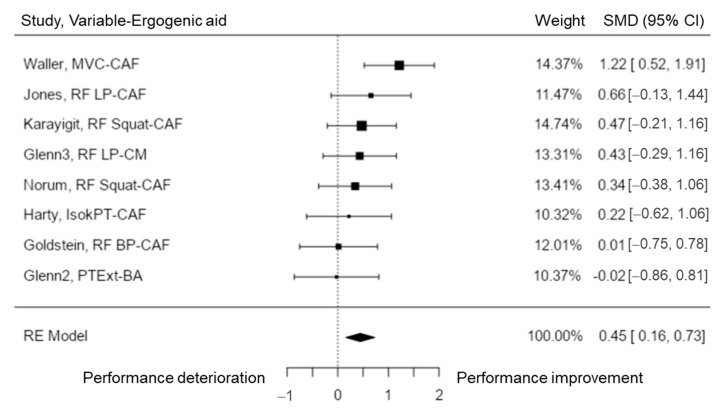
Resistance strength performance forest plot. MVC = maximal voluntary contraction; RF = repetitions until failure; LP = leg press; PT = peak torque; Isok = isokinetic; Ext = extension.

To analyze resistance strength performance, eight studies [40,44,45,47,50,51,60,72] were included in the analysis. The observed SMD ranged from −0.02 to 1.22, with most of the estimates being positive (88%). The estimated average SMD according to the random-effects model was \hat{\mu} = 0.45 (95% CI: 0.16 to 0.73). Hence, the average outcome was significantly different from zero (z = 3.05, *p* = 0.002). According to the Q-test, there was no presence of a significant amount of heterogeneity in the true outcomes (Q (7) = 7.80, *p* = 0.350, tau^2^ = 0.02, I^2^ = 13.89%). A 95% prediction interval for the true outcomes varied between 0.03 and 0.86. Consequently, although there may be some heterogeneity, the true outcomes of the studies are generally in the same direction as the estimated average outcome. The analysis of the studentized residuals showed that none of the studies had a value larger than ± 2.73 and this model did not show outliers. None of the studies could be considered to be overly influential as explained by the Cook’s distances (Figure 4). Neither the rank correlation nor the regression test indicated any funnel plot asymmetry (*p* = 0.275 and *p* = 0.100, respectively). Hence, the results indicated that CAF may help to enhance the number of repetitions until failure in lower body exercises.

#### 3.1.2. Sprint

**Figure 5:**
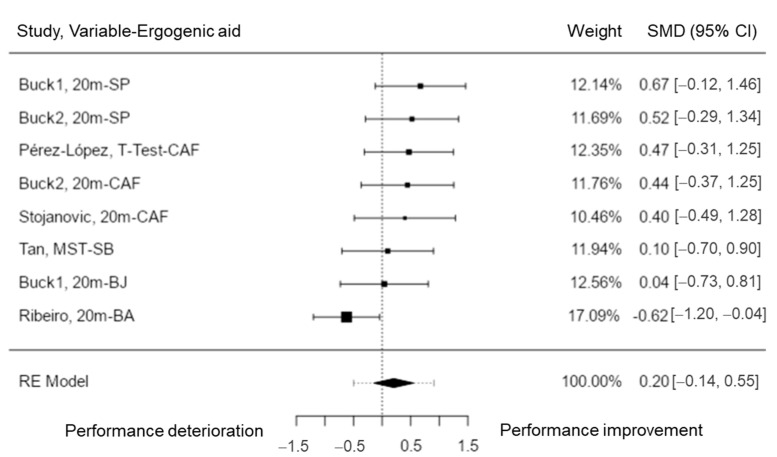
Sprint performance forest plot. 20m = 20 m sprint; MST = mean sprint time; T-Test = T Agility Test.

Sprint capacity was assessed in six studies [29,52,58,63,73,75], with a total of k = 8 variables included in the analysis. The observed SMD presented values between −0.62 and 0.67, with the majority of estimates being positive (88%). The estimated average SMD based on the random-effects model was \hat{\mu} = 0.21 (95% CI: −0.14 to 0.55). Therefore, the average outcome was significantly different from zero (z = 1.15, *p* = 0.248). Based on the Q-test, the true outcomes showed no significant amount of heterogeneity (Q (7) = 10.85, *p* = 0.145, tau^2^ = 0.10, I^2^ = 38.76%). A 95% prediction interval for the true outcomes ranged between −0.50 and 0.91. Thus, even though the average outcome is taken to be positive, in some studies the true outcome may in fact be negative. The studentized residuals revealed that one variable (Ribeiro, 20 m-BA) [63] may be a potential outlier in the context of this model due to a value higher than ± 2.73. In addition, based on the Cook’s distances, this variable could be considered to be overly influential (Figure 5). The regression test indicated funnel plot asymmetry (*p* = 0.003) but not the rank correlation test (*p* = 0.275). Therefore, the results showed that both SP and CAF help to improve sprint performance.

#### 3.1.3. Cardiorespiratory Fitness

- Aerobic Capacity

**Figure 6:**
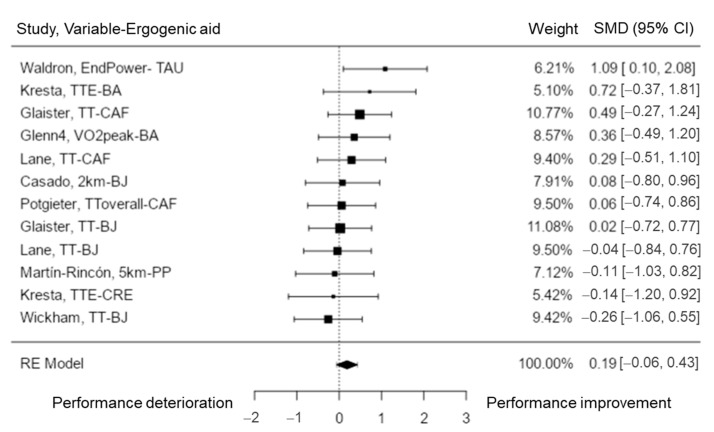
Aerobic performance forest plot. TTE = time to exhaustion; TT = time trial.

Aerobic capacity was analyzed in nine studies [28,54,55,56,59,64,68,74,77], including a total of k = 12 variables. The observed SMD varied from −0.26 to 1.09, with most estimates being positive (67%). The estimated average SMD based on the random-effects model was \hat{\mu} = 0.19 (95% CI: −0.06 to 0.43). Thus, the average outcome was significantly different from zero (z = 1.48, *p* = 0.140). The Q-test revealed no significant amount of heterogeneity in the true outcomes (Q (11) = 7.50, *p* = 0.757, tau^2^ = 0.00, I^2^ = 0.00%). The studentized residuals analysis showed that none of the variables had a value larger than ± 2.87 and thus there was no manifestation of outliers in the context of this analysis. None of the variables could be overly influential according to the Cook’s distances (Figure 6). Neither the rank correlation nor the regression test demonstrated any funnel plot asymmetry (*p* = 0.545 and *p* = 0.398, respectively). Thus, the results showed that TAU could enhance end power, while CAF could help to improve time trial performance, and time to exhaustion could be extended by BA consumption. No conclusive effects of BJ, PP, or CRE in improving aerobic performance were shown.

- Anaerobic capacity

**Figure 7:**
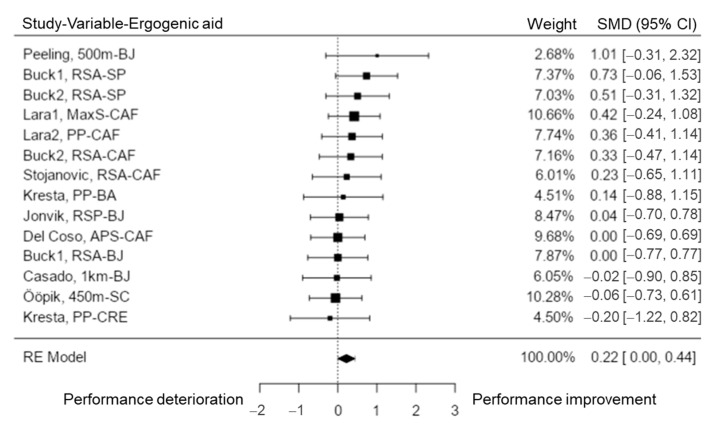
Anaerobic performance forest plot. RSA = repeated sprint ability; MaxS = maximal speed; PP = peak power; RSP = repeated sprint performance; APS = average peak speed.

This capacity was analyzed in 11 studies [10,28,29,46,53,58,64,69,70,73,76], including a total of k = 14 variables. The observed SMD ranged from −0.20 to 1.01, with most estimates being positive (64%). The estimated average SMD based on the random-effects model was \hat{\mu} = 0.22 (95% CI: 0.00 to 0.44). Thus, the average outcome was significantly different from zero (z = 2.00, *p* = 0.05). The Q-test revealed no significant amount of heterogeneity in the true outcomes (Q (13) = 6.59, *p* = 0.922, tau^2^ = 0.00, I^2^ = 0.00%). The studentized residuals revealed that none of the variables had a value larger than ± 2.91 and this model was not affected by outliers. None of the variables could be considered to be overly influential based on the Cook’s distances (Figure 7). Neither the rank correlation nor the regression test indicated any funnel plot asymmetry (*p* = 0.518 and *p* = 0.528, respectively). Therefore, the results showed that SP and CAF could help to improve repeated sprint performance. No conclusive results could be obtained from the other EA. 

### 3.2. Risk of Bias Assessment

Of the 32 studies included in the quantitative analysis, 31 obtained scores of 7 to 8, considered as “good” according to the PEDro tool (Table 2). The exception was the study carried out by Peeling et al. [69], in which the participants were five elite kayak athletes, where there was no randomization process nor blinding of the investigators. The score of 8 considered as “optimal” was given to 17 of the 32 articles [10,29,39,40,43,44,45,47,51,52,58,59,64,70,73,74,75]. The point for the blinding of subjects and assessors was awarded to those who specified that the study was “double blind” although they did not explain the procedure in the methodology. Of the 32 articles, 14 described the study sample but not the established inclusion criteria [29,43,46,52,53,54,55,56,63,69,70,74,75,76]. Only two articles [59,70] specified the initial number of participants and the final number from which the results were obtained, being more than 85% of the first sample. According to the statistical analysis, 17 articles did not provide effect sizes of the outcomes [10,46,50,53,54,55,56,59,60,63,68,69,70,71,72,76,77]. 

## 4. Discussion

This systematic review with meta-analysis shows a comprehensive analysis of the efficacy of different EAs on sports performance in female athletes and recreational practitioners. Specifically, it analyzed which types of EA were the most used to improve each physical capacity and how they affected the different performance tests. The results showed that plenty of EAs are utilized, although the use of CAF predominates over the rest.

The meta-analysis carried out on strength capacity, specifically power performance, showed CAF as the most used EA (in eight of the nine articles included). The variables that manifested the biggest improvement when taking this EA were those related to jumping performance, specifically in the CMJ test, and to the velocity of upper limb resistance exercises. This result coincides with the study carried out by Grgic [14] where CAF ingestion impacted a wide array of outcomes during the CMJ test in male athletes. In addition, peak velocity in an upper limb resistance exercise seemed to be enhanced with the use of CAF. Velocity in resistance exercise has also been improved after the ingestion of CAF in other studies, such as those reported in the review of Raya-Gonzalez et al. [79]. Regarding the dose of CAF used, most studies used doses of 3 mg/kg or 6 mg/kg, which showed performance improvements in both males and females [43,80,81,82]. However, a lower dose of 0.9 to 2 mg/kg has also been shown to enhance mean velocity, muscular endurance, and muscular strength [83]. 

As for isometric strength, CAF was also the most utilized EA. This EA helped to increase isometric peak torque, which has been demonstrated in other studies in men to date [84]. As for hand grip strength, CAF seemed to enhance this variable as well, although most of the studies proving its effects have been carried out in the male population [85].

The most used EA to improve resistance strength performance was CAF, which helped to improve repetitions until failure in lower body exercises. Something similar is shown in the literature, although most studies in men focus on bench presses or upper body exercises to measure this parameter [86,87,88]. As for BA, no significant results can be drawn regarding the effects of this EA on strength enhancement in women. In most of the studies concerning the ingestion of BA, aerobic and anaerobic capacities are affected in terms of delaying the onset of fatigue, which allows athletes to perform longer or more intense training sessions [89]. Its effect on strength has only been measured in a few studies, most with males [90,91]. In the study of Outlaw et al. [92], muscular endurance was assessed after an eight-week protocol of resistance training supplemented with BA in a novice college female population. Muscular endurance improved, but with similar effects in the BA and placebo group, suggesting that the enhancement came from the training protocol itself rather than the BA consumption. 

In terms of improving sprint performance, heterogeneity of EAs has been found. Those that seem to be most effective are SP and CAF. The effects of CAF on sprint performance in female athletes are similar to the improvements found in men [82,93]. As for SP, the hypothesis behind its consumption is that increased phosphate content can enhance the rate of ATP and PCr resynthesis [94]. So far, there are no studies measuring the effects of this EA on isolated sprint improvement. The studies to date measure the effects on repeated sprinting ability [58,73,95,96,97]. 

In terms of aerobic performance, a variety of EAs were used. Due to this heterogeneity of EAs, hardly any consistent effects were shown, so no conclusive data can be drawn as to which EA would be better for use by female athletes. The use of TAU to improve end power [74], BA to reduce fatigue [64] when performing incremental exercise tests, and CAF in 20 km cycling time trials [55] stand out slightly. 

Something similar occurs with anaerobic performance, where no studies show hugely significant results. The use of BJ in the study of Peeling et al. [69] showed better results. However, due to the small sample size and the high risk of bias, these data cannot be taken with confidence. For the improvement of repeated sprint ability, SP and CAF stand out slightly [29,58,73].

Something to point out is that one of the most used EAs to improve repeated sprint ability is CRE, with several published articles testing its efficacy in male athletes, mostly with positive effects [96,98,99,100,101,102]. However, for women there are only three articles that study its effects on anaerobic performance [65,66,67]. In these articles, the effects of CRE ingestion are measured with respect to a placebo in female soccer or futsal players. In the three studies, the improvement of performance in sprinting, jumping, and anaerobic power is shown. However, in the study by Ramírez-Campillo et al., CRE consumption is integrated with a plyometric training program. Both the placebo and the CRE group significantly improved in comparison to the control group that did not perform plyometrics or take any EA. The CRE group obtained better results than the placebo group for peak jump power, squat jump performance, and mean sprint time variables, although it is difficult to assess if this was due to the CRE itself or to a greater adaptation to plyometric training. 

Taking this into account, more studies measuring the effect of CRE on anaerobic capacity in female athletes are needed, leaving a gap in the scientific literature that needs to be addressed.

To our understanding, this is the first systematic review with meta-analysis conducted on the use of EAs in female athletes, bringing together all the existing studies which analyze their impact on exercise performance. It is therefore a first step towards future interventions, showing where there is a gap in scientific knowledge. 

The limitations of this study include the great heterogeneity found in terms of the type of EA used. Another limitation is the variety of different exercise tests for physical capacities, whereby due to the methodological differences of the studies, it has not been possible to perform a meta-analysis of the specific variables. Furthermore, something to note is the difference sporting ability of the athletes included in the studies, whereby the effects on performance may be different due to variations in training load and expertise level.

However, with this study we wanted to show which EAs are the most studied to date to improve strength, sprint, or cardiorespiratory capacity and in which specific tests they show better results. This information can be used by coaches and athletes to guide their choice in deciding which EA to take to improve exercise performance.

## 5. Conclusions

CAF is shown to be the ergogenic aid most commonly used by female athletes. It was found to be the best EA to improve strength performance, specifically jumping capacity, isometric strength values, and repetitions until failure.

Regarding sprinting and speed, CAF and SP are the EAs that show the best results. The positive effects of CRE that have been demonstrated in males cannot be proven for females due to the absence of well-conducted studies carried out in women.

As for cardiorespiratory fitness capacity, aerobic test results could be improved with the use of TAU, CAF, and BA. No conclusive effects of BJ, PP, or CRE in improving aerobic performance were shown. In terms of anaerobic variables, both CAF and SP could help to improve repeated sprint ability.

Nevertheless, more studies are needed in female athletes that measure the effects of different EAs on sports performance, such as BJ, BA, or SP, as studies to date are scarce and there are many types of EA that need to be further considered in this population, such as CRE and TAU.

## Figures and Tables

**Table 1:** Characteristics of the studies included in the systematic review.

[Author, Journal—Year]	Title	Aim	Sample (*n*), age, BMI	EA-Dosis	Effects on	Conclusions
[Martín-Rincón, Nutrients—2020]	Supplementation with a Mango Leaf Extract (Zynamite®) in Combination with Quercetin Attenuates Muscle Damage and Pain and Accelerates Recovery after Strenuous Damaging Exercise	To examinate if Zynamite administered in combination with quercetin, interfere or not with muscle performance and promotes recovery after repeated damaging exercise	Students of sports sciences (48), 23.3, 22.2	PP 140 mg of Zynamite + 140 mg of quercetin (1 h before and every 8 h thereafter for 24 h)	5 km running 10 km race followed by 100 drop jumps	140 mg Zynamite + 140 mg consumption might be effective to decrease muscle pain and damage, and to boost muscle performance recovery
[Waldron, Amino Acids—2018]	The effects of taurine on repeat sprint cycling after low or high cadence exhaustive exercise in females	To investigate the effects of TAU vs. PLA on RSA, performed after fixed incremental ramp exercise to exhaustion in women	University lacrosse players (9), 22, 24.5	TAU Acute 50 mg/kg	6 × 10 s sprint in cycle ergometer (90 r/min or 30 r/min) Heart rate (HR) Blood lactate	TAU decreased HR before exercise and enhanced end-test power output, detrimentally affecting sprint performance, regardless of cadence. Short endurance performance may improve acutely following TAU intake, but this effect may not be sustained over longer exercise periods or urge the need for longer recovery periods.
[Wickham, Physiol. Rep.—2019]	No effect of beetroot juice supplementation on exercise economy and performance in recreationally active females despite increased torque production	To explore if acute and chronic BJ ingestion reduced de O2 cost of submaximal exercise, improved aerobic TT performance and improved skeletal muscle torque production in recreationally active females.	Recreationally active (12; 12); 23; 22; 22.8; 23	BJ 6.5 mmol nitrate 2 times/day (8 days)	Study 1: VO_2_ at 50/70 VO_2_peak Study 2: MVC plantar flexors	BJ did not decrease submaximal exercise VO_2_ nor increase aerobic TT performance in recreationally active women.
[Waller, PeerJ—2020]	A low caffeine dose improves maximal strength, but not relative muscular endurance in either heavier-or lighter-loads, or perceptions of effort or discomfort at task failure in females	To determine the effects of a 100 mg CAF dose on maximal voluntary contraction (MVC) and time to task failure (TTF) at different loads in active women	Active (19), 21.6, 24.2	CAF Acute 100 mg	MVC knee extensors 70% MVC and 30% MVC load isometric knee extension TTF RPE RPD	A 100 mg dose of CAF showed positive effects in knee extension MVC, although the significance of this performance improvement is less clear. Statistically significant effects of 100 mg dose of CAF on TTF, RPD and RPE were not found.
[Kresta, JISSN—2012]	Effects of 28 days of beta-alanine and creatine monohydrate supplementation on muscle carnosine, body composition and exercise performance in recreationally active females	To determine the acute and long-term effects of BA, alone or combined with CRE, on muscle carnosine and phosphagen levels, aerobic and anaerobic exercise performance and body composition in recreationally active women	Recreationally active (32)	BA: 0.1 g/kg/day CR: 0.3 g/kg/day week 1, 0.1 g/kg/day week 2–4 (28 days)	Aerobic exercise capacity: VO_2_peak, TTE, METS, Ventilatory threshold WANT Blood lactate Body composition Muscle carnosine and phospagen levels	The combination of BA and CRE ingestion did not produce any consistent additive benefits in recreationally active females.
[Smith, Amino Acids—2012]	Exercise-induced oxidative stress: the effects of b-alanine supplementation in women	To evaluate the effects of BA supplementation on markers of oxidative stress	Moderate active (24), 21.7, 22.7	BA 800 mg/tablet, 2 tablets 3 times/day (28 days)	VO_2_max 40-min treadmill run at baseline 70% PV	The long-term effects of BA ingestion indicated little benefits on aerobic performance variables and little antioxidant potential in active females.
[Ramírez-Campillo, JSAMS—2015]	Effects of plyometric training and creatine supplementation on maximal-intensity exercise and endurance in female soccer players	To explore the effects of a 6-week intervention program of plyometric training and CRE ingestion on maximal intensity and endurance performance in female soccer players.	Amateur soccer players (30), 22.9, 22.6	CRE 20 g/d 1 week 5 g/d 5 week (6 weeks)	Jumping performance Reactive strength index RAST 20 m sprint 20 m multi stage shuttle run test	Adaptations to plyometric training may be enhanced with CRE supplementation
[Buck1, Eur. J. Appl. Physiol.—2015]	Effects of sodium phosphate and beetroot juice supplementation on repeated-sprint ability in females	To investigate the effects of SP and BJ (alone and combined) on repeated-sprint ability (RSA) in female team sport players	Amateur team sport players (netball, basketball, soccer) (13), 25.5, 22.2	SP: 50 mg/kg 4 times/day (6 days) BJ: 6 mmol nitrate	Team-game circuit 4 × 15 min 6 × 20 m RSA	SP ingestion enhanced RSA in team sport women athletes before, midway and after of a STGC.
[Buck2, J. Sports Sci.—2015]	Effects of sodium phosphate and caffeine loading on repeated-sprint ability	To explore the effect of CAF and SP ingestion (alone or in combination) on RSA performance in women	Amateur team sport players (netball, basketball, soccer) (12), 25.5, 22.2	SP: 550 mg/kg 4 times/day (6 days) CAF: 6 mg/kg	Team-game circuit 4 × 15 min 6 × 20 m RSA	SP and SP and CAF enhanced RSA in fresh and fatiguing conditions (Set 1, 2 and 3)
[Casado, Appl. Sciences—2021]	Influence of Sex and Acute Beetroot Juice Supplementation on 2 km Running Performance	To address the effect of acute nitrate-rich BJ ingestion on 2 km running performance in amateur long distance runners and to investigate possible different responses according to sex	Amateur long distance runners (10), 36.6, 21.2	BJ Acute 12.8 mmol nitrate	2 km time trial (TT)	Acute BJ intake enhances the second half of a 2 km running TT and decreases RPE in men and women athletes.
[Filip-Stachnik, Eur. J. Nutr.—2022]	Acute effects of two caffeine doses on bar velocity during the bench press exercise among women habituated to caffeine: a randomized, crossover, double-blind study involving control and placebo conditions	To determine the efficacy of two different doses of CAF to improve bar velocity during the bench press exercise in women habituated to CAF	Recreational resistance trained athletes (12), 23.3, 24.1	CAF Acute 6 mg/kg 3 mg/kg	3 × 3 reps of bench press 50% 1RM	6 mg/kg of CAF may be an effective ergogenic dose to enhance power-training outcomes in women used to CAF consumption. However, the effects of this CAF dose was only superior to the control condition, not to the PLA. This may indicate that the effects may be produced by performance expectation and biological parameters.
[Ali1, J. Sports Sci.—2015]	Caffeine ingestion enhances perceptual responses during intermittent exercise in female team-game players	To investigate the effect of CAF ingestion in female team sport athletes using OCS during and following an intermittent exercise protocol on cognitive performance, perceptual responses and mood state	Recreational to international team sport athletes (soccer, hockey, netball) (10), 24, 22.7	CAF Acute 6 mg/kg	Cognitive performance POMS RPE, FAS, FS Blood measures	CAF intake enhanced vigour and showed a tendency to reduce fatigue on perceptual parameters during an intermittent running exercise.
[Ali2, JISSN—2016]	The influence of caffeine ingestion on strength and power performance in female team-sport players	To determine the influence of acute evening CAF ingestion during and following intermittent exercise on knee extensor and flexor strength and power in female team-sport athletes	Recreational to international team sport athletes (soccer, hockey, netball) (10), 24	CAF Acute 6 mg/kg	Blood measures Muscle torque and power CMJ	The ingestion of CAF improved power and eccentric strength in team sport athletes during intermittent running protocol as well as during the following morning.
[Lara1, Amino Acids—2014]	Caffeine-containing energy drink improves physical performance in female soccer players	To examinate the effects of an energy drink containing CAF to enhance physical performance during a simulated game in female soccer players	Soccer players (18), 21, 22.3	CAF Acute 3 mg/kg	CMJ 7 × 30 m sprint Soccer match 2 × 40 min	The intake of a 3 mg/ kg CAF energy drink improved CMJ hight, RSA, total running distance, the distance covered and sprint velocity during a simulated game. Therefore, energy drinks containing CAF could be useful to raise physical performance in female football players
[Glenn3, Eur. J. Nutr.—2017]	Acute citrulline malate supplementation improves upper- and lower-body submaximal weightlifting exercise performance in resistance-trained females	To explore effects of acute CM ingestion on upper and lower body weightlifting performance in resistance-trained athletes.	Resistance trained athletes (15), 23, 25.4	CM Acute 8 g	1 RM for the bench press and leg press. 6 sets to failure (80% 1 RM)	Acute CM ingestion in resistance trained athletes improved bench press and leg press exercise performance and decreased RPE during bench press exercise. The acute intake of CM may potentially enhance performance in muscular endurance-based sports.
[Harty, Front. Nutr.—2020]	Caffeine Timing Improves Lower-Body Muscular Performance: A Randomized Trial	To discover the optimal pre-exercise time to ingest CAF to enhance lower-body muscular performance and to identify the presence of any possible sex differences in responses to pre-exercise time CAF ingestion.	Resistance trained athletes (11), 20.1, 24.2	CAF Acute 6 mg/kg	Isometric mid-thigh pull test, CMJ Isometric knee extension test Isokinetic knee extensor fatigue protocol	1 h pre-exercise CAF intake presented the most significant ergogenic effects comparing to other time points. Thus, 1 h is the recommended time point to consume CAF when peak muscular performance is wanted.
[Lara2, BJCP—2019]	Acute caffeine intake increases performance in the 15-s Wingate test during the menstrual cycle	To determine the effects of CAF ingestion on Wingate anaerobic test (WANT) performance along 3 phases of de menstrual cycle	Eumenorrhoeic triathletes (13), 31, 21.3	CAF Acute 3 mg/kg	15 s WANT	A similar effect of CAF on WANT peak power was found in the follicular, preovulatory and midluteal phases. Therefore, CAF might be an effective ergogenic aid by enhancing anaerobic performance during the different phases of the menstrual cycle in eumenorrhoeic athletes.
[Sahl Abad, BJHPA—2021]	Purslane supplementation lowers oxidative stress, inflammatory and muscle damage biomarkers after high-intensity intermittent exercise in female runners	To assess the effect of 10-day PUR ingestion on oxidative stress inflammation and muscle damage biomarkers after completing HIIE	Runners (9), 23, 19	PUR 1000 mg (500 mg/2 times/day) (10 days)	Blood measures	10 days of PUR suplementation decreased the biomarkers perturbation of muscle damage, oxidative stress and inflammation.
[Romero-Moraleda, Nutrients—2019]	The Effect of Caffeine on the Velocity of Half-Squat Exercise during the Menstrual Cycle: A Randomized Controlled Trial	To assess the effect of acute CAF intake on mean and peak velocity of half-squat exercise during three different phases of the menstrual cycle	Eumenorrhoeic trained athletes (running, cycling, swimming) (13), 31, 21.3	CAF Acute 3 mg/kg	Half-squat maximal velocity (20%, 40% 60% and 80% of 1 RM)	During the 3 phases of the menstrual cycle, the positive ergogenic effects of CAF were similar.
[Karayigit, Sports—2021]	Combined but Not Isolated Ingestion of Caffeine and Taurine Improves Wingate Sprint Performance in Female Team-Sport Athletes Habituated to Caffeine	To examine the effect of CAF + TAU ingestion during WANT exercise	Team sport athletes (rugby, football, basketball) (17), 23.4, 21.1	CAF: 6 mg/kg TAU 1 g	WANT	The combined ingestion of CAF and TAU enhanced both PP and MP in team sport athletes used to CAF consumption. CAF and TAU alone did not improve WANT performance.
[Jones, Nutrients—2021]	The Dose-Effects of Caffeine on Lower Body Maximal Strength, Muscular Endurance, and Rating of Perceived Exertion in Strength-Trained Females	To determine the effect that CAF ingestion has on strength performance and RF on resistance trained athletes	Resistance trained athletes (14), 23, 23.6	CAF Acute 6 mg/kg 3 mg/kg	1 RM leg press Repetitions to failure (RF) 60% 1 RM Total weigth lifted (TV) RPE	Doses of 3 mg/kg and 6 mg/kg of CAF increased muscular endurance in resistance trained women. Higher doses of CAF did not cause further statistical significance in muscular endurance.
[Rosas, J. Hum. Kinet.—2017]	Effects of Plyometric Training and Beta-Alanine Supplementation on Maximal Intensity Exercise and Endurance in Female Soccer Players	To examine the effects of a 6-week plyometric training program and its combination with BA supplementation, on endurance and maximal intensity performance in soccer players	Soccer players (25), 23.7, 22.2	BA 4.8 g/day (6 weeks)	Single and repeated jumps and sprints, endurance, and change-of-direction speed performance	Implementation of a 6-week plyometric training protocol enhanced jumping, sprinting and endurance performance in female soccer players. The consumption of BA improved the effects of plyometric training on the endurance running outcomes, jumping abilities and repeated sprinting.
[Ööpik, JSSM—2008]	The effects of sodium citrate ingestion on metabolism and 1500-m racing time in trained female runners	To explore the effects of SC ingestion in a 1500-m run competition in middle-distance runners	Middle-distance runners (17), 18.6, 19.9	SC Acute 0.4 g/kg	1500 m Body mass Blood measurements	SC intake produces an increase in water retention, plasma volume and body mass, as well as moderates a rise in blood glucose concentration during exercise. It does not enhance 1500-m running performance in middle-distance women runners.
[Glaister, JSCR—2015]	Effects of dietary nitrate, caffeine, and their combination on 20 km cycling time-trial performance	To investigate the effects of acute BJ and CAF (alone or combined) on 20 km cycling time-trial performance	Cyclists and triathletes (14), 31, 21.6	BJ: 0.45 g nitrate CAF: 5 mg/kg	Power output Distance completed Cadence VO_2_ max RER (respiratory exchange ratio) RPE HR Blood measurements	CAF ingestion demonstrates the beneficial effects on endurance performance. Acute ingestion of CAF combined with BJ adds nothing to the benefits given by CAF consumption.
[Harmancı, TOJRAS—2013]	Effects of Creatine Supplementation on Motor Performance in Female Futsal Players	To determine the effects of 15-day CRE ingestion on motor performance of female futsal players	Futsal players (28), 21.2, 20.7	CRE 4 × 5 g 5 days 5 g 10 days (15 days)	Grip strength Back Strength 20 m sprint Jumping measurements WANT	The CRE ingestion protocol presented enhanced speed, agility and explosive strength outcomes in female futsal players.
[Goldstein, JISSN—2010]	Caffeine enhances upper body strength in resistance-trained women	To examine the impact of CAF intake on strength and muscular endurance in resistance trained athletes	Resistance trained athletes (15), 24.6, 23	CAF Acute 6 mg/kg	1RM bench press RF 60% 1 RM	CAF ingestion in a moderate dose may be effective for improving muscular performance in resistance trained athletes.
[Norum, Scand. J. Med. Sci. Sports—2019]	Caffeine increases strength and power performance in resistance-trained females during early follicular phase	To investigate, the effects of 4 mg/kg CAF consumed during the early follicular phase on strength and power variables in resistance trained athletes	Resistance trained athletes (15), 29.8, 23.2	CAF Acute 4 mg/kg	CMJ MVC (right knee extensor muscles) 1 RM squat and bench press RF 60% 1 RM RPE Plasma analysis	The 4 mg/kg intake of CAF improved muscular endurance, power and maximal strength in resistance trained women used to CAF consumption, during the early follicular phase. Few adverse effects were found.
[Potgieter, IJSNEM—2017]	Caffeine improves triathlon performance:a field studyin males and females	To elucidate the effect of CAF on triathlon event performance using a field study design, while allowing investigation into potential mechanisms at play	Triathlon athletes (12), 37.2, 21.3	CAF Acute 6 mg/kg	TT RPE POMS Blood lactate	The ingestion 6 mg/kg of CAF, 45–60 minutes before the beginning of a Olympic-distance triathlon is recommended to enhance performance.
[Lane, Appl. Physiol. Nutr. Metab.—2014]	Single and combined effects of beetroot juice and caffeine supplementation on cycling time trial performance	To determine the combined effects of BJ and CAF ingestion on a cycling time trial (TT) simulating the 2012 London Olympic Games course	Cyclists and triathletes (12), 28, 21.7	CAF: 3 mg/kg BJ: 8.4 mmol nitrate	29.35 km course on a cycle ergometer	A 3 mg/kg dose CAF administered in a gum improved cycling TT performance. BJ ingestion did not produce any ergogenic effect in this study.
[Stojanovic, Appl. Physiol. Nutr. Metab.—2018]	Acute caffeine supplementation promotes small to moderate improvements in performance tests indicative of in-game success in professional female basketball players	To assess the effect of acute CAF ingestion on anaerobic performance in elite female basketball players	Elite basketball players (10), 20.2, 22.6	CAF Acute 3 mg/kg	CMJ CMJ with arm swing Squat jump Lane Agility Drill, 20 m sprints 20 m sprints dribbling a ball Suicide Run.	Acute intake of CAF could produce small to moderate enhancements in basketball performance variables as well as reducing RPE.
[Karayigit, Nutrients—2021]	Effects of Different Doses of Caffeinated Coffee on Muscular Endurance, Cognitive Performance, and Cardiac Autonomic Modulation in Caffeine Naive Female Athletes	To assess the effect of CAF ingestion on lower-upper body muscular endurance, cognitive performance, and heart rate variability (HRV) in female team sport athletes	Elite resistance trained team sport athletes (rugby, handball, soccer) (17), 23, 22.7	CAF Acute 6 mg/kg 3 mg/kg	Heart rate variability RF 40% 1 RM squat and bench press Cognitive performance	The ingestion of both 3 mg/kg and 6 mg/kg of CAF improved lower body muscular endurance and cognitive performance without generating an extra cardiovascular load.
[Atakan, Sci. Sports—2019]	Short term creatine loading without weight gain improves sprint, agility and leg strength performance in female futsal players	To assess the effects of 7-day CRE ingestion on lower-body strength, agility and velocity in elite futsal players	Elite futsal players (30), 19.8, 20.8	CRE 0.25 g/kg/day (7 days)	Velocity (10, 20, 30 m sprints) Leg strength Illinois agility test Body weight (BW)	0.25 gr/kg/d of CRE consumption during 7 days is helpful for enhancing performance in elite female futsal players with no additional effects in BW.
[Tan, Int. J. Sport Nutr. Exerc. Metab.—2010]	Effects of Induced Alkalosis on Simulated Match Performance in Elite Female Water Polo Players	To determine the effects of acute SB (NaHCO_3_) ingestion on simulated water polo match performance	Elite waterpolo players (12), 23.7, 25.3	SB Acute 0.3 g/kg	56 × 10 maximal sprint swims	SB ingestion did not produce substantial improvement on intermittent-sprint performance in waterpolo players
[Portillo, JSCR—2017]	Effects of caffeine ingestion on skill performance during an international female rugby sevens competition	To assess the effects of an energy drink containing CAF on technical performance in elite rugby players	Elite rugby players (16), 23, 24	CAF Acute 3 mg/kg	Body impacts Technical actions: tackle, ruck, pass, pass receive, ball carry	A dose of 3 mg/kg of CAF contained in an energy drink, may produce a higher engagement of the rugby players during a game due to and increment of the number of body impacts. The CAF intake did not influence the quality nor the frequency of any specific technical actions during the game.
[Muñoz, IJSPP—2020]	Effects of Caffeine Ingestion on Physical Performance in Elite Women Handball Players: A Randomized, Controlled Study	To assess the effects of acute CAF consumption on exercise performance in elite handball players	Elite handball players (15), 22.6, 24	CAF Acute 3 mg/kg	CMJ Ball throws Isometric handgrip strength Agility T test Sprint 30 m 2 × 20 min handball match	3 mg/kg of CAF intake increased sprint and jump performance, ball-throwing velocity, and in-game frequency accelerations and decelerations in elite female handball players.
[Peeling, IJSNEM—2015]	Beetroot Juice Improves On-Water 500 m Time-Trial Performance, and Laboratory-Based Paddling Economy in National and International-Level Kayak Athletes	To explore the effects of BJ intake in kayak athletes in field-based settings	Elite kayak athletes (5), 25, 23.7	BJ Acute 9.6 mmol nitrate	500 m on-water kayak time trial RPE Blood lactate	The ingestion of BJ produced a significant enhancement on TT during a field-based 500 m kayak trial.
[Pérez-López, MSSE—2015]	Caffeinated Energy Drinks Improve Volleyball Performance in Elite Female Players	To assess the impact of a CAF-containing energy drink on exercise performance in volleyball players	Elite volleyball players (13), 25.2, 21.3	CAF Acute 3 mg/kg	Jumping parameters Manual dynamometry Agility T-test	Energy drinks containing CAF may enhance physical performance in female volleyball players and thus the accuracy during an actual volleyball match.
[Del Coso, Amino Acids—2013]	Caffeine-containing energy drink improves sprint performance during an international rugby sevens competition	To examine the effects of a CAF-containing energy drink on exercise performance during a rugby sevens game	Elite rugby players (16), 23, 24	CAF Acute 3 mg/kg	15-s maximal jump test 6 × 30 m RSA Rugby games: running pace, instantaneous speed RPE	Women’s rugby players enhanced their physical performance during a game due to the intake of 3 mg/kg of CAF contained in an energy drink.
[Ribeiro, Front. Nutr.—2020]	Short-duration beta-alanine supplementation did no tprevent the detrimental effects of an intense preparatory period on exercise capacity in top-level female footballers	To determine the impact of 3-week BA supplementation during a football-specific training period on high-intensity running performance variables	Elite football players (24), 18, 22.5	BA 6.4 g/day (3 weeks)	YoYo Intermittent Recovery Test Level 1 (YoYo IR1) RAST 20 m sprint	Elite female football players participating in a 3-week intense training period worsened their high-intensity intermittent exercise capacity. BA ingestion during the same period did not attenuated this negative result.
[Jonvik, IJSNEM—2018]	The Effect of Beetroot Juice Supplementationon Dynamic Apnea and Intermittent Sprint Performance in Elite Female Water Polo Players	To investigate the effects of BJ supplementation on dynamic apnea and RSA in elite water polo players	Elite waterpolo players (14), 22, 23	BJ 800 mg/day nitrate (6 days)	Dynamic apnea test 16 × 15 m RSA (4 × 4 block)	BJ consumption does not enhance intermittent performance in elite female water polo players, but may produce a potential ergogenic effect during dynamic apnea.
[Glenn1, EJSS—2016]	Acute citrulline-malate supplementation improves maximal strength and anaerobic power in female, masters athletes tennis players	To investigate the effects of acute CM ingestion on vertical power, grip strength, and anaerobic cycling performance in female, master tennis players	Masters athletes tennis players (17), 51, 23.7	CM Acute 8 g	Grip strength, Vertical power WANT	Maximal grip strength and anaerobic cycling power could be enhanced by a 8 g consumption of CM in female MA. The intake of CM before competition might improve tennis performance in this population.
[Glenn2, JSCR—2016]	Effects of 28-day beta-alanine supplementation in isokinetic exercise performance and body composition in female masters athletes	To determine the effects of a 28-day BA supplementation on ISO, handgrip strength (HG), and body composition in female master cyclists	Master cyclists (22), 53, 25.1	BA 800 mg 4 times/day (28 days)	Isometric grip Isokinetic strenght analysis Body composition	28-days of BA consumption improved peak torque values and work completed. Thus, BA enhances lower-body exercise performance in MA women.
[Glenn4, Amino Acids—2015]	Incremental effects of 28 days of beta-alanine supplementation on high-intensity cycling performance and blood lactate in masters female cyclists	To investigate the effects of a 28-day ingestion of BA on lactate clearance, total work completed and time to exhaustion in master cyclists women	Master cyclists (22), 53.3, 25	BA 800 mg 4 times/day (28 days)	Time to exhaustion (TTE) at 120% VO_2_max Total work completed (TWC) Blood lactate	The BA ingestion during 28 days improved TTE and TWC on cycling with additional lactate clearance during passive rest in mater cyclists women

**Table 2:** Risk of bias scores according to the PEDro tool.

Study	Items										Total Score
	1	2	3	4	5	6	7	8	9	10	
Casado	*	*	*	*	*	*	*	*	*	*	7/10
Kresta	*	*	*	*	*	*	*	*	*	*	8/10
Martín-Rincón	*	*	*	*	*	*	*	*	*	*	7/10
Wickham	*	*	*	*	*	*	*	*	*	*	7/10
Jones	*	*	*	*	*	*	*	*	*	*	8/10
Glaister	*	*	*	*	*	*	*	*	*	*	7/10
Ööpik	*	*	*	*	*	*	*	*	*	*	7/10
Tan	*	*	*	*	*	*	*	*	*	*	8/10
Waller	*	*	*	*	*	*	*	*	*	*	8/10
Karayigit	*	*	*	*	*	*	*	*	*	*	8/10
Ali	*	*	*	*	*	*	*	*	*	*	8/10
Harty	*	*	*	*	*	*	*	*	*	*	8/10
Filip-Stachnik	*	*	*	*	*	*	*	*	*	*	8/10
Stojanovic	*	*	*	*	*	*	*	*	*	*	8/10
Waldron	*	*	*	*	*	*	*	*	*	*	8/10
Buck1	*	*	*	*	*	*	*	*	*	*	8/10
Buck2	*	*	*	*	*	*	*	*	*	*	8/10
Lara1	*	*	*	*	*	*	*	*	*	*	7/10
Lara2	*	*	*	*	*	*	*	*	*	*	8/10
Glenn1	*	*	*	*	*	*	*	*	*	*	7/10
Glenn2	*	*	*	*	*	*	*	*	*	*	7/10
Glenn3	*	*	*	*	*	*	*	*	*	*	7/10
Glenn4	*	*	*	*	*	*	*	*	*	*	8/10
Jonvik	*	*	*	*	*	*	*	*	*	*	8/10
Lane	*	*	*	*	*	*	*	*	*	*	7/10
Potgietier	*	*	*	*	*	*	*	*	*	*	7/10
Ribeiro	*	*	*	*	*	*	*	*	*	*	7/10
Del Coso	*	*	*	*	*	*	*	*	*	*	7/10
Pérez-López	*	*	*	*	*	*	*	*	*	*	8/10
Peeling	*	*	*	*	*	*	*	*	*	*	4/10
Norum	*	*	*	*	*	*	*	*	*	*	8/10
Goldstein	*	*	*	*	*	*	*	*	*	*	7/10

1 = subjects were randomly allocated to groups (in a crossover study, subjects were randomly allocated an order in which treatments were received); 2 = allocation was concealed; 3 = the groups were similar at baseline regarding the most important prognostic indicators; 4 = there was blinding of all subjects; 5 = there was blinding of all therapists who administered the therapy; 6 = there was blinding of all assessors who measured at least one key outcome; 7 = measures of at least one key outcome were obtained from more than 85% of the subjects initially allocated to groups; 8 = all subjects for whom outcome measures were available received the treatment or control condition as allocated or, where this was not the case, data for at least one key outcome was analyzed by “intention to treat”; 9 = the results of between-group statistical comparisons are reported for at least one key outcome; 10 = the study provides both point measures and measures of variability for at least one key outcome [35]; * = Yes; * = No.

## Data Availability

Not applicable.

## References

[1] Garthe I., Maughan R.J. (2018). Athletes and Supplements: Prevalence and Perspectives. Int. J. Sport Nutr. Exerc. Metab..

[2] Piccardi N., Manissier P. (2009). Nutrition and nutritional supplementation. Impact on skin health and beauty. Derm. Endocrinol..

[3] Porrini M., del Bo C. (2016). Ergogenic Aids and Supplements. Front. Horm. Res..

[4] Gepner Y., Varanoske A.N., Boffey D., Hoffman J.R. (2019). Benefits of β-hydroxy-β-methylbutyrate supplementation in trained and untrained individuals. Res. Sports Med..

[5] Salehi M., Mashhadi N.S., Esfahani P.S., Feizi A., Hadi A., Askari G. (2021). The Effects of Curcumin Supplementation on Muscle Damage, Oxidative Stress, and Inflammatory Markers in Healthy Females with Moderate Physical Activity: A Randomized, Double Blind, Placebo Controlled Clinical Trial. Int. J. Prev. Med..

[6] Barry D.W., Hansen K.C., Van Pelt R., Witten M., Wolfe P., Kohrt W.M. (2011). Acute Calcium Ingestion Attenuates Exercise-Induced Disruption of Calcium Homeostasis. Med. Sci. Sports Exerc..

[7] Close G., Hamilton D., Philp A., Burke L., Morton J. (2016). New strategies in sport nutrition to increase exercise performance. Free Radic. Biol. Med..

[8] Stout J.R., Cramer J.T., Zoeller R.F., Torok D., Costa P., Hoffman J.R., Harris R.C., O’kroy J. (2007). Effects of b-alanine supplementation on the onset of neuromuscular fatigue and ventilatory threshold in women. Amino Acids.

[9] McMahon N.F., Leveritt M.D., Pavey T.G. (2017). The Effect of Dietary Nitrate Supplementation on Endurance Exercise Performance in Healthy Adults: A Systematic Review and Meta-Analysis. Sports Med..

[10] Lara B., Hellín J.G., Ruiz C., Romero-Moraleda B., Del Coso J. (2020). Acute caffeine intake increases performance in the 15-s Wingate test during the menstrual cycle. Br. J. Clin. Pharmacol..

[11] Burke L.M. (2019). Supplements for Optimal Sports Performance. Curr. Opin. Physiol..

[12] Peeling P., Binnie M.J., Goods P.S., Sim M., Burke L.M. (2018). Evidence-Based Supplements for the Enhancement of Athletic Performance. Int. J. Sport Nutr. Exerc. Metab..

[13] Glenn J.M., Gray M., Gualano B., Roschel H. (2016). The Ergogenic Effects of Supplemental Nutritional Aids on Anaerobic Performance in Female Athletes. Strength Cond. J..

[14] Grgic J., Mikulic P., Schoenfeld B.J., Bishop D.J., Pedišić Ž. (2019). The Influence of Caffeine Supplementation on Resistance Exercise: A Review. Sports Med..

[15] Eckerson J.M. (2016). Creatine as an Ergogenic Aid for Female Athletes. Strength Cond. J..

[16] Sawicka A.K., Renzi G., Olek R.A. (2020). The bright and the dark sides of L-carnitine supplementation: A systematic review. J. Int. Soc. Sports Nutr..

[17] Somerville V., Bringans C., Braakhuis A. (2017). Polyphenols and Performance: A Systematic Review and Meta-Analysis. Sports Med..

[18] Díaz M.S., Martín-Castellanos A., Fernández-Elías V.E., Torres O.L., Calvo J.L. (2022). Effects of Polyphenol Consumption on Recovery in Team Sport Athletes of Both Sexes: A Systematic Review. Nutrients.

[19] Khan N., Mukhtar H. (2019). Tea Polyphenols in Promotion of Human Health. Nutrients.

[20] Luca S.V., Macovei I., Bujor A., Miron A., Skalicka-Woźniak K., Aprotosoaie A.C., Trifan A. (2020). Bioactivity of dietary polyphenols: The role of metabolites. Crit. Rev. Food Sci. Nutr..

[21] Pechanova O., Dayar E., Cebova M. (2020). Therapeutic Potential of Polyphenols-Loaded Polymeric Nanoparticles in Cardiovascular System. Molecules.

[22] Moreno B., Veiga S., Sánchez-Oliver A.J., Domínguez R., Morencos E. (2022). Analysis of Sport Supplement Consumption by Competitive Swimmers According to Sex and Competitive Level. Nutrients.

[23] Sánchez-Oliver A.J., Domínguez R., López-Tapia P., Tobal F.M., Jodra P., Montoya J.J., Guerra-Hernández E.J., Ramos-Álvarez J.J. (2021). A Survey on Dietary Supplement Consumption in Amateur and Professional Rugby Players. Foods.

[24] Muñoz A., López-Samanes Á., Domínguez R., Moreno-Pérez V., Sánchez-Oliver A.J., Del Coso J. (2020). Use of Sports Supplements in Competitive Handball Players: Sex and Competitive Level Differences. Nutrients.

[25] Domínguez R., López-Domínguez R., López-Samanes Á., Gené P., González-Jurado J.A., Sánchez-Oliver A.J. (2020). Analysis of Sport Supplement Consumption and Body Composition in Spanish Elite Rowers. Nutrients.

[26] Mata F., Domínguez R., López-Samanes Á., Sánchez-Gómez Á., Jodra P., Sánchez-Oliver A.J. (2021). Analysis of the consumption of sports supplements in elite fencers according to sex and competitive level. BMC Sports Sci. Med. Rehabil..

[27] Knapik J.J., Steelman R.A., Hoedebecke S.S., Austin K.G., Farina E.K., Lieberman H.R. (2016). Prevalence of Dietary Supplement Use by Athletes: Systematic Review and Meta-Analysis. Sports Med..

[28] Casado A., Domínguez R., Fernandes Da Silva S., Bailey S.J. (2021). Influence of Sex and Acute Beetroot Juice Supplementation on 2 KM Running Performance. Appl. Sci..

[29] Stojanović E., Stojiljković N., Scanlan A.T., Dalbo V.J., Stanković R., Antić V., Milanović Z. (2019). Acute caffeine supplementation promotes small to moderate improvements in performance tests indicative of in-game success in professional female basketball players. Appl. Physiol. Nutr. Metab..

[30] Soldin O.P., Mattison D. (2009). Sex Differences in Pharmacokinetics and Pharmacodynamics. Clin. Pharmacokinet..

[31] Gandhi M., Aweeka F., Greenblatt R.M., Blaschke T.F. (2004). Sex Differences in Pharmacokinetics and Pharmacodynamics. Annu. Rev. Pharmacol. Toxicol..

[32] Temple J.L., Ziegler A.M. (2011). Gender Differences in Subjective and Physiological Responses to Caffeine and the Role of Steroid Hormones. J. Caffeine Res..

[33] Branch J.D. (2003). Effect of Creatine Supplementation on Body Composition and Performance: A Meta-analysis. Int. J. Sport Nutr. Exerc. Metab..

[34] Stegen S., Bex T., Vervaet C., Vanhee L., Achten E., Derave W. (2014). β-Alanine Dose for Maintaining Moderately Elevated Muscle Carnosine Levels. Med. Sci. Sports Exerc..

[35] Cashin A.G., McAuley J.H. (2020). Clinimetrics: Physiotherapy Evidence Database (PEDro) Scale. J. Physiother..

[36] Viechtbauer W. (2010). Conducting Meta-Analyses in R with the metafor Package. JSS J. Stat. Softw..

[37] Cochran W.G. (1954). The Combination of Estimates from Different Experiments. Biometrics.

[38] The Jamovi Project [Computer Software]. http://www.jamovi.org.

[39] Filip-Stachnik A., Krzysztofik M., Del Coso J., Wilk M. (2022). Acute effects of two caffeine doses on bar velocity during the bench press exercise among women habituated to caffeine: A randomized, crossover, double-blind study involving control and placebo conditions. Eur. J. Nutr..

[40] Harty P.S., Zabriskie H.A., Stecker R.A., Currier B.S., Tinsley G.M., Surowiec K., Jagim A.R., Richmond S.R., Kerksick C.M. (2020). Caffeine Timing Improves Lower-Body Muscular Performance: A Randomized Trial. Front. Nutr..

[41] Romero-Moraleda B., Del Coso J., Gutiérrez-Hellín J., Lara B. (2019). The Effect of Caffeine on the Velocity of Half-Squat Exercise during the Menstrual Cycle: A Randomized Controlled Trial. Nutrients.

[42] Ali A., O’Donnell J., Von Hurst P., Foskett A., Holland S., Starck C., Rutherfurd-Markwick K. (2016). Caffeine ingestion enhances perceptual responses during intermittent exercise in female team-game players. J. Sports Sci..

[43] Ali A., O’Donnell J., Foskett A., Rutherfurd-Markwick K. (2016). The influence of caffeine ingestion on strength and power performance in female team-sport players. J. Int. Soc. Sports Nutr..

[44] Karayigit R., Naderi A., Akca F., Da Cruz C.J.G., Sarshin A., Yasli B.C., Ersoz G., Kaviani M. (2020). Effects of Different Doses of Caffeinated Coffee on Muscular Endurance, Cognitive Performance, and Cardiac Autonomic Modulation in Caffeine Naive Female Athletes. Nutrients.

[45] Jones L., Johnstone I., Day C., Le Marquer S., Hulton A.T. (2021). The Dose-Effects of Caffeine on Lower Body Maximal Strength, Muscular Endurance, and Rating of Perceived Exertion in Strength-Trained Females. Nutrients.

[46] Lara B., González-Millán C., Salinero J.J., Abián-Vicén J., Areces F., Barbero-Alvarez J.C., Muñoz V., Portillo L.J., Rave J.M.G., Del Coso J. (2014). Caffeine-containing energy drink improves physical performance in female soccer players. Amino Acids.

[47] Waller G., Dolby M., Steele J., Fisher J.P. (2020). A low caffeine dose improves maximal strength, but not relative muscular endurance in either heavier-or lighter-loads, or perceptions of effort or discomfort at task failure in females. PeerJ.

[48] Portillo J., Del Coso J., Abián-Vicén J. (2017). Effects of Caffeine Ingestion on Skill Performance During an International Female Rugby Sevens Competition. J. Strength Cond. Res..

[49] Muñoz A., López-Samanes A., Pérez-López A., Aguilar-Navarro M., Moreno-Heredero B., Rivilla-García J., González-Frutos P., Pino-Ortega J., Morencos E., Del Coso J. (2020). Effects of Caffeine Ingestion on Physical Performance in Elite Women Handball Players: A Randomized, Controlled Study. Int. J. Sports Physiol. Perform..

[50] Goldstein E., Jacobs P.L., Whitehurst M., Penhollow T., Antonio J. (2010). Caffeine enhances upper body strength in resistance-trained women. J. Int. Soc. Sports Nutr..

[51] Norum M., Risvang L.C., Bjørnsen T., Dimitriou L., Rønning P.O., Bjørgen M., Raastad T. (2020). Caffeine increases strength and power performance in resistance-trained females during early follicular phase. Scand. J. Med. Sci. Sports.

[52] Pérez-López A., Salinero J.J., Abián-Vicén J., Valadés D., Lara B., Hernandez C., Areces F., González C., Del Coso J. (2015). Caffeinated Energy Drinks Improve Volleyball Performance in Elite Female Players. Med. Sci. Sports Exerc..

[53] Del Coso J., Portillo J., Muñoz G., Abián-Vicén J., Gonzalez-Millán C., Muñ oz-Guerra J. (2013). Caffeine-containing energy drink improves sprint performance during an international rugby sevens competition. Amino Acids.

[54] Potgieter S., Wright H.H., Smith C. (2018). Caffeine Improves Triathlon Performance: A Field Study in Males and Females. Int. J. Sport Nutr. Exerc. Metab..

[55] Glaister M., Pattison J.R., Muniz-Pumares D., Patterson S., Foley P. (2015). Effects of Dietary Nitrate, Caffeine, and Their Combination on 20-km Cycling Time Trial Performance. J. Strength Cond. Res..

[56] Lane S.C., Hawley J.A., Desbrow B., Jones A.M., Blackwell J.R., Ross M.L., Zemski A.J., Burke L.M. (2014). Single and combined effects of beetroot juice and caffeine supplementation on cycling time trial performance. Appl. Physiol. Nutr. Metab..

[57] Karayigit R., Naderi A., Saunders B., Forbes S.C., Del Coso J., Berjisian E., Yildirim U.C., Suzuki K. (2021). Combined but Not Isolated Ingestion of Caffeine and Taurine Improves Wingate Sprint Performance in Female Team-Sport Athletes Habituated to Caffeine. Sports.

[58] Buck C., Guelfi K., Dawson B., McNaughton L., Wallman K. (2015). Effects of sodium phosphate and caffeine loading on repeated-sprint ability. J. Sports Sci..

[59] Glenn J.M., Gray M., Stewart R., Moyen N.E., Kavouras S.A., DiBrezzo R., Turner R., Baum J. (2015). Incremental effects of 28 days of beta-alanine supplementation on high-intensity cycling performance and blood lactate in masters female cyclists. Amino Acids.

[60] Glenn J.M., Gray M., Stewart R.W., Moyen N.E., Kavouras S.A., Dibrezzo R.O., Turner R., Baum J.I., Stone M.S. (2016). Effects of 28-Day Beta-Alanine Supplementation on Isokinetic Exercise Performance and Body Composition in Female Masters Athletes. J. Strength Cond. Res..

[61] Smith A.E., Stout J.R., Kendall K.L., Fukuda D.H., Cramer J.T. (2012). Exercise-induced oxidative stress: The effects of b-alanine supplementation in women. Amino Acids.

[62] Rosas F., Ramírez-Campillo R., Martínez C., Caniuqueo A., Cañas-Jamet R., McCrudden E., Meylan C., Moran J., Nakamura F.Y., Pereira L.A. (2017). Effects of Plyometric Training and Beta-Alanine Supplementation on Maximal-Intensity Exercise and Endurance in Female Soccer Players. J. Hum. Kinet..

[63] Ribeiro R., Duarte B., Da Silva A.G., Ramos G.P., Picanço A.R., Penna E.M., Coswig V., Barbalho M., Gentil P., Gualano B. (2020). Short-Duration Beta-Alanine Supplementation Did Not Prevent the Detrimental Effects of an Intense Preparatory Period on Exercise Capacity in Top-Level Female Footballers. Front. Nutr..

[64] Kresta J.Y., Oliver J.M., Jagim A.R., Fluckey J., Riechman S., Kelly K., Meininger C., Mertens-Talcott S.U., Rasmussen C., Kreider R.B. (2014). Effects of 28 days of beta-alanine and creatine supplementation on muscle carnosine, body composition and exercise performance in recreationally active females. J. Int. Soc. Sports Nutr..

[65] Ramírez-Campillo R., González-Jurado J.A., Martínez C., Nakamura F.Y., Peñailillo L., Meylan C.M., Caniuqueo A., Cañas-Jamet R., Moran J., Alonso-Martínez A.M. (2016). Effects of plyometric training and creatine supplementation on maximal-intensity exercise and endurance in female soccer players. J. Sci. Med. Sport.

[66] Atakan M.M., Karavelioğlu M.B., Harmancı H., Cook M., Bulut S. (2019). Short term creatine loading without weight gain improves sprint, agility and leg strength performance in female futsal players. Sci. Sports.

[67] Harmancı H., Kalkavan A., Karavelioğlu M.B., Şentürk A. (2013). Effects of Creatine Supplementation on Motor Performance in Female Futsal Players. Online J. Recreat. Sport.

[68] Wickham K.A., McCarthy D.G., Pereira J.M., Cervone D.T., Verdijk L., van Loon L.J., Power G.A., Spriet L.L. (2019). No effect of beetroot juice supplementation on exercise economy and performance in recreationally active females despite increased torque production. Physiol. Rep..

[69] Peeling P., Cox G., Bullock N., Burke L.M. (2015). Beetroot Juice Improves On-Water 500 M Time-Trial Performance, and Laboratory-Based Paddling Economy in National and International-Level Kayak Athletes. Int. J. Sport Nutr. Exerc. Metab..

[70] Jonvik K.L., van Dijk J.-W., Senden J.M., van Loon L.J., Verdijk L.B. (2018). The Effect of Beetroot Juice Supplementation on Dynamic Apnea and Intermittent Sprint Performance in Elite Female Water Polo Players. Int. J. Sport Nutr. Exerc. Metab..

[71] Glenn J.M., Gray M., Jensen A., Stone M.S., Vincenzo J.L. (2016). Acute citrulline-malate supplementation improves maximal strength and anaerobic power in female, masters athletes tennis players. Eur. J. Sport Sci..

[72] Glenn J.M., Gray M., Wethington L.N., Stone M.S., Stewart R.W., Moyen N.E. (2017). Acute citrulline malate supplementation improves upper- and lower-body submaximal weightlifting exercise performance in resistance-trained females. Eur. J. Nutr..

[73] Buck C.L., Henry T., Guelfi K., Dawson B., Mc Naughton L.R., Wallman K. (2015). Effects of sodium phosphate and beetroot juice supplementation on repeated-sprint ability in females. Eur. J. Appl. Physiol..

[74] Waldron M., Knight F., Tallent J., Patterson S., Jeffries O. (2018). The effects of taurine on repeat sprint cycling after low or high cadence exhaustive exercise in females. Amino Acids.

[75] Tan F., Polglaze T., Cox G., Dawson B., Mujika I., Clark S. (2010). Effects of induced alkalosis on simulated match performance in elite female water polo players. Int. J. Sport Nutr. Exerc. Metab..

[76] Oöpik V., Timpmann S., Kadak K., Medijainen L., Karelson K. (2008). The Effects of Sodium Citrate Ingestion on Metabolism and 1500-m Racing Time in Trained Female Runners. J. Sports Sci. Med..

[77] Martin-Rincon M., Gelabert-Rebato M., Galvan-Alvarez V., Gallego-Selles A., Martinez-Canton M., Lopez-Rios L., Wiebe J.C., Martin-Rodriguez S., Arteaga-Ortiz R., Dorado C. (2020). Supplementation with a Mango Leaf Extract (Zynamite^®^) in Combination with Quercetin Attenuates Muscle Damage and Pain and Accelerates Recovery after Strenuous Damaging Exercise. Nutrients.

[78] Abad A.C.S., Gram A., Soori R., Akbarnejad A., Ghuchan F.A., Zare M.M., Hackney A.C. (2021). Purslane supplementation lowers oxidative stress, inflammatory and muscle damage biomarkers after high-intensity intermittent exercise in female runners. Balt. J. Health Phys. Act..

[79] Raya-González J., Rendo-Urteaga T., Domínguez R., Castillo D., Rodríguez-Fernández A., Grgic J. (2020). Acute Effects of Caffeine Supplementation on Movement Velocity in Resistance Exercise: A Systematic Review and Meta-analysis. Sports Med..

[80] Ouergui I., Mahdi N., Delleli S., Messaoudi H., Chtourou H., Sahnoun Z., Bouassida A., Bouhlel E., Nobari H., Ardigò L.P. (2022). Acute Effects of Low Dose of Caffeine Ingestion Combined with Conditioning Activity on Psychological and Physical Performances of Male and Female Taekwondo Athletes. Nutrients.

[81] Chen H.-Y., Wang H.-S., Tung K., Chao H.-H. (2015). Effects of Gender Difference and Caffeine Supplementation on Anaerobic Muscle Performance. Endoscopy.

[82] López-Samanes Á., Moreno-Pérez V., Travassos B., Del Coso J. (2021). Effects of acute caffeine ingestion on futsal performance in sub-elite players. Eur. J. Nutr..

[83] Grgic J. (2022). Exploring the minimum ergogenic dose of caffeine on resistance exercise performance: A meta-analytic approach. Nutrition.

[84] Venier S., Grgic J., Mikulic P. (2019). Caffeinated Gel Ingestion Enhances Jump Performance, Muscle Strength, and Power in Trained Men. Nutrients.

[85] Grgic J. (2022). Effects of caffeine on isometric handgrip strength: A meta-analysis. Clin. Nutr. ESPEN.

[86] Davis J.-K., Green M., Laurent M. (2011). Effects of Caffeine on Resistance Training Performance on Repetitions to Failure. Med. Sci. Sports Exerc..

[87] Duncan M.J., Oxford S. (2011). The Effect of Caffeine Ingestion on Mood State and Bench Press Performance to Failure. J. Strength Cond. Res..

[88] Karayigit R., Koz M., Sánchez-Gómez A., Naderi A., Yildirim U.C., Domínguez R., Gur F. (2021). High Dose of Caffeine Mouth Rinse Increases Resistance Training Performance in Men. Nutrients.

[89] Murphy M.J., Rushing B.R., Sumner S.J., Hackney A.C. (2022). Dietary Supplements for Athletic Performance in Women: Beta-Alanine, Caffeine, and Nitrate. Int. J. Sport Nutr. Exerc. Metab..

[90] Roveratti M.C., Jacinto J.L., Oliveira D.B., da Silva R.A., Andraus R.A.C., de Oliveira E.P., Ribeiro A.S., Aguiar A.F. (2019). Effects of beta-alanine supplementation on muscle function during recovery from resistance exercise in young adults. Amino Acids.

[91] Maté-Muñoz J.L., Lougedo J.H., Garnacho-Castaño M.V., Veiga-Herreros P., Lozano-Estevan M.D.C., García-Fernández P., de Jesús F., Guodemar-Pérez J., Juan A.F.S., Domínguez R. (2018). Effects of β-alanine supplementation during a 5-week strength training program: A randomized, controlled study. J. Int. Soc. Sports Nutr..

[92] Outlaw J.J., Smith-Ryan A.E., Buckley A.L., Urbina S.L., Hayward S., Wingfield H.L., Campbell B., Foster C., Taylor L.W., Wilborn C.D. (2016). Effects of β-Alanine on Body Composition and Performance Measures in Collegiate Women. J. Strength Cond. Res..

[93] Raya-González J., Scanlan A.T., Soto-Célix M., Rodríguez-Fernández A., Castillo D. (2021). Caffeine Ingestion Improves Performance During Fitness Tests but Does Not Alter Activity During Simulated Games in Professional Basketball Players. Int. J. Sports Physiol. Perform..

[94] Buck C.L., Wallman K.E., Dawson B., Guelfi K.J. (2013). Sodium Phosphate as an Ergogenic Aid. Sports Med..

[95] Stein J.A., Gasier H.G., Goodman B.D., Ramirez M.R., Delatorre B.P., Beattie C.M., Barstow T.J., Heinrich K.M. (2021). Effects of Caffeine on Exercise Duration, Critical Velocity, and Ratings of Perceived Exertion During Repeated-Sprint Exercise in Physically Active Men. Int. J. Exerc. Sci..

[96] Glaister M., Howatson G., Abraham C.S., Lockey R.A., Goodwin J.E., Foley P., Mcinnes G. (2008). Caffeine Supplementation and Multiple Sprint Running Performance. Med. Sci. Sports Exerc..

[97] Schneiker K.T., Bishop D., Dawson B., Hackett L.P. (2006). Effects of Caffeine on Prolonged Intermittent-Sprint Ability in Team-Sport Athletes. Med. Sci. Sports Exerc..

[98] Peyrebrune M.C., Nevill M.E., Donaldson F.J., Cosford D.J. (2011). The effects of oral creatine supplementation on performance in single and repeated sprint swimming. J. Sports Sci..

[99] Skare O.-C., Skadberg Ø., Wisnes A.R. (2001). Creatine supplementation improves sprint performance in male sprinters. Scand. J. Med. Sci. Sports.

[100] Vandebuerie F., Eynde B.V., Vandenberghe K., Hespel P. (1998). Effect of Creatine Loading on Endurance Capacity and Sprint Power in Cyclists. Endoscopy.

[101] Aaserud R., Gramvik P., Olsen S.R., Jensen J. (1998). Creatine supplementation delays onset of fatigue during repeated bouts of sprint running. Scand. J. Med. Sci. Sports.

[102] Izquierdo M., Ibañez J., González-Badillo J.J., Gorostiaga E.M. (2002). Effects of creatine supplementation on muscle power, endurance, and sprint performance. Med. Sci. Sports Exerc..

